# Olfactory Neuroblastoma—A Challenging Fine Line between Metastasis and Hematology

**DOI:** 10.3390/medicina59040731

**Published:** 2023-04-08

**Authors:** Trandafir Cornelia Marina, Balica Nicolae Constantin, Baderca Flavia, Sarau Oana Silvana, Poenaru Marioara, Cristian Andrei Sarau

**Affiliations:** 1 ENT Department, Spitalul Clinic Municipal de Urgenta, Victor Babeş University of Medicine and Pharmacy, Bulevardul. Revolutiei No. 6, 300054 Timisoara, Romania; 2 ENT Department, Victor Babeş University of Medicine and Pharmacy, 300041 Timişoara, Romania; 3 Department of Microscopic Morphology, Victor Babeş University of Medicine and Pharmacy, 300041 Timişoara, Romania; 4 Department of Hematology, Victor Babeş University of Medicine and Pharmacy, 300041 Timisoara, Romania; 5 Department of Medical Semiology I, Victor Babeş University of Medicine and Pharmacy, 300041 Timişoara, Romania

**Keywords:** olfactory neuroblastoma, rare sinonasal tumors, endoscopic surgery, management strategy, myeloma

## Abstract

Developing in a limited space, rare tumors located at the nose and paranasal sinuses are sometimes difficult to diagnose due to their modest clinical presentation, which is uncorrelated with anatomopathological diversity. This limits the preoperative diagnosis without added immune histochemical study; for that reason, we present our experience with these tumors with the intention of raising awareness. The patient included in our study was investigated by our department through clinical and endoscopic examination, imaging investigations, and an anatomic-pathological study. The selected patient gave consent for participation and inclusion in this research study in compliance with the 1964 Declaration of Helsinki.

## 1. Introduction

Tumors in the nose and paranasal sinuses are rare lesions, affecting <1 in 100,000 people per year [1]. These tumors may have monomorphic, nonspecific symptomatology, but they require a prompt and accurate diagnosis due to their poor prognosis and evolution.

While new discoveries are made worldwide, especially to assess new methods of prevention, diagnosis, or specialized treatment, the rarity of these cases remains an obstacle in choosing the management option.

Olfactory neuroblastoma, also referred to as esthesioneuroblastoma, represents an oncological entity of neuroectodermal origin arising in the upper part of the nasal cavity. Its incidence represents 2–3% of all nasal neoplasms [2].

The etiology and risk factors are still unknown. As with most sinonasal tumors, the symptoms are nonspecific, most commonly epistaxis, nasal obstruction, and hyposmia. A biopsy is essential for diagnosis, while CT scans and MRI images are used for the staging system.

These tumors are locally aggressive, with the propensity to spread into the anterior skull base as well as to metastasize to the cervical lymph nodes, thorax, and bones [3].

Cervical metastases are described in 5–8% of cases at diagnosis and in 15–25% of patients as recurrence [4,5]. The management of the neck in olfactory neuroblastoma is still controversial.

In our day, three separate staging systems exist. Kadish et al. [6] proposed in 1976 a staging of olfactory neuroblastoma in three groups based on the extension of the disease, which is still widely used (group A—tumor limited to the nasal fossa; group B—extension to the paranasal sinuses; group C—extension beyond the paranasal sinuses). The staging has evolved, and a modified Kadish system was proposed with an additional group D describing tumors with the locoregional or distant presence of metastases. Some institutions apply the TNM staging system by the American Joint Committee on Cancer (AJCC) based on the Dulguerov modified version of staging [6,7] (Table 1).

There is no agreed-upon standard treatment for olfactory neuroblastoma. Surgical treatment and radiotherapy represent the most frequently used approaches. Chemotherapy treatment modalities can be used in selected cases. Open surgical approaches, such as extracranial and anterior craniofacial resection, are preferred in the treatment of ONB. The development of an endoscopic approach over the past few decades has gained popularity and offered many advantages (better cosmetic outcome and better visualization of some deep areas within the sinonasal region), representing a valid treatment for olfactory neuroblastoma [4,8].

Multimodal treatment associating surgery with radiation therapy has been demonstrated to have the best survival rates; however, the infrequency of olfactory neuroblastoma and its heterogeneous clinical biology limit the possibility of creating specific protocols of treatment [3,9,10,11].

Recurrence may be encountered years after treatment; therefore, long-term follow-up is recommended [9].

Multiple myeloma currently affects 250,000 people globally [12]. According to the 2022 Canadian data, the incidence is 10.1 per 100,000 men and 6.4 per 100,000 women, with an average age of diagnosis in the 6thdecade for both [13].

The diagnosis is made based on the evidence of one or more of the CRAB criteria (C—hypercalcemia, R—renal insufficiency, A—anemia, and B—bone lesions), with biopsy confirmation of bone marrow infiltration by 10% clonal plasma cells or detection of a plasmacytoma. The laboratory diagnosis includes a variety of biochemical examinations (serum protein electrophoresis, urine protein electrophoresis, urine immunofixation, serum free light chains, and total protein) associated with the monitoring of end-organ damage [14,15].

## 2. Case Report

A 45-year-old patient was admitted to our department with epistaxis from the right nasal fossa. He received conservative treatment using an anterior nasal tampon (Merocel) for 2 days, and he returned a few days later with recurrent symptomatology. The patient underwent an endoscopic examination, where he presented a solitary, smooth mass in the right nasal fossa.

**Figure 1:**
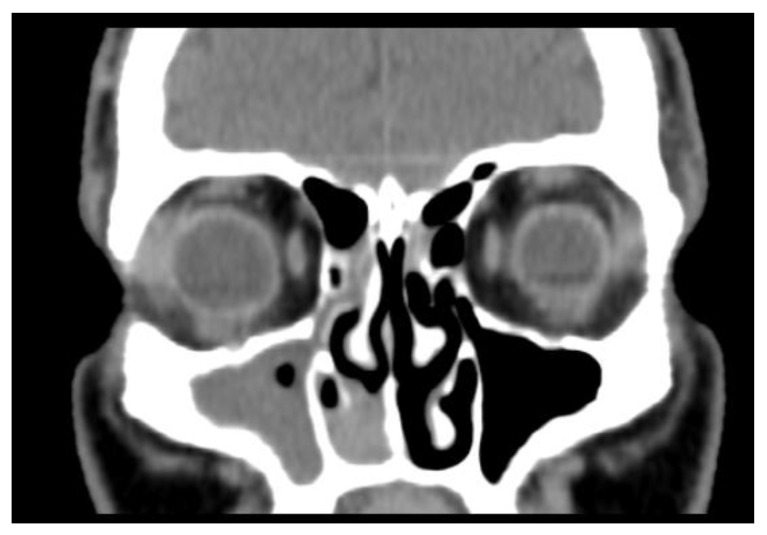
Preoperative CT scan showing a nonspecific opacification of the right nasal fossa (inferior meatus) with partial erosion of the inferior turbinate. There was no evidence of invasion into the skull base, cribriform plate, or olfactory cleft. The CT scan demonstrated density and mucosal thickening of the maxillary sinus.

The patient underwent CT and MRI of the head and neck prior to surgical resection; the CT scan identified a heterogeneous, natively hyperdense tissue mass, located at the level of the right nasal cavity, which it filled for the most part. The mass-produced remodeling and bone erosion, with partial visualization of the right inferior nasal turbinates. The native CT appearance was nonspecific in the differential diagnosis, including an inverted papilloma, sinonasal polyp, or adenocarcinoma. The right maxillary sinus was filled with mixed, parafluid, and tissue densities (Figure 1).

The patient underwent a chest and abdominal CT scan and MRI, and there was no proof of distant metastasis. The patient was staged according to the Kadish system.

The tumor was located in the nasal fossa, without intracranial extension or erosion of the cribriform plate. An endoscopic approach was performed. The tumor was successfully removed with negative margins; a right maxillary antrostomy was performed. A Merocel was placed in the nasal fossa and removed after 48 h. In order to avoid the risk of infection, for all endoscopic surgeries, we use an antibioprophylaxis for 48–72 h, initiated on the day of the surgery.

The patient had no complications (no CSF leak, no neurologic symptoms), and no epistaxis was found after the postoperative Merocel removal. The patient stayed in the hospital for less than a week.

Tissue fragments were immunohistologically studied; the fragments were initially fixed in formalin. Positive and negative controls were performed.

The tumor consisted of a polypoid thickened respiratory mucosa through a pseudolobular tumor proliferation that develops exclusively in the lamina propria, respecting the structure of the covering epithelium. Tumor cells were relatively monomorphic, small in size, weakly basophilic or weakly acidophilic, with a nucleus with irregularly or finely dispersed chromatin and inconstant nucleoli; some cells have a central nucleus, and in other cells, the position of the nucleus is eccentric, rare mitoses (1–2 cp × 40), with solid and trabecular growth architecture in a fibrous stroma and the formation of pseudorosettes (Homer Wright rosettes), and frequently in perivascular position, encasing small and medium vessels.Positive and negative controls were performed.

Immunohistochemical reactions are performed on the paraffin block. The negative immunoreactions in tumor cells were identified (CD45, AE1/AE3, CD34, SMA, desmina, and CD99). BCL-2 was positive, and PGP9.5 was positive.

**Figure 2:**
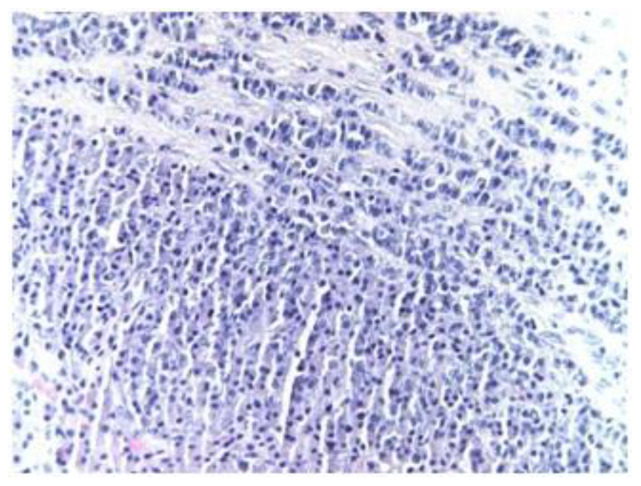
Olfactory neuroblastoma (HE stain, 40×) showing a solid and trabecular growth pattern composed of round, small cells that are relatively uniform with scanty cytoplasm and scattered chromatin pattern.

**Figure 3:**
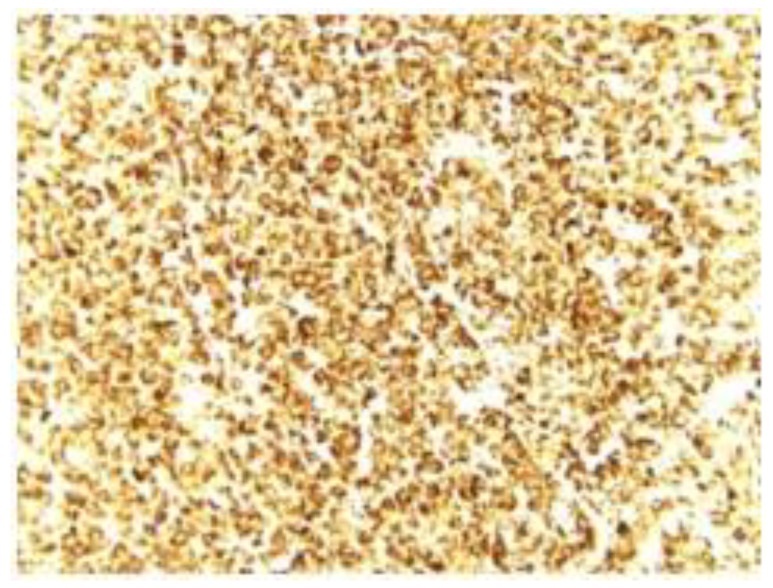
Immunohistochemical stain (PGP 9.5, ob 40×) showing intense cytoplasmic positive reaction of PGP 9.5 in all tumor cells.

Correlating the histological aspects (Figure 2) with immunohistochemical staining, the diagnosis of neuroectodermal tumor was established, with the subtype of olfactory neuroblastoma (BCL2-positive and PGP9.5-positive) determined at G2 differentiation (Figure 3).

After the surgical endoscopic removal of the tumor and the confirmation of the diagnosis by the anatomopathological exam, the patient was referred for radiotherapy. He underwent 33 sessions of radiotherapy treatment (DT = 50 Gy/fr/38 days) on the right nasal fossa. Radiotherapy was well tolerated.

Follow-up was conducted by the oncology and radiotherapy departments, where postoperative imaging of the head and neck was performed. No recurrence was identified. The ENT follow-up was scheduled every 3 months during the first year for nasal endoscopy, every 6 months during the second and third years, and annually thereafter without recurrence or symptoms.

In the fifth year after the primary diagnosis, the patient complained of lumbosacral and humeral bone pain.

He underwent a CT scan and an MRI, which indicated osteolytic lesions of the vertebral bodies C6, C7, C8, T7 (16 mm), and T11, as well as the costal arches and bone basin (11 cm at the level of the right iliac wing with the extension to the right iliac muscle and a 10 mm lesion at the left iliac wing). The CT scan identified an important circumferential thickening of the mucosa of the right maxillary sinus that almost completely occupied it, with otherwise normally aerated paranasal sinuses.

The lesions raised suspicion of multiple myeloma or long-distance metastasis. A biopsy of the sinus mucosa was performed with local anesthesia; the patient was referred to a hematologist and scheduled for a whole-body positron emission computer tomography (PET-CT) and a bone biopsy. The sinus biopsy revealed hypertrophic mucosa and the absence of tumoral cells.

**Figure 4:**
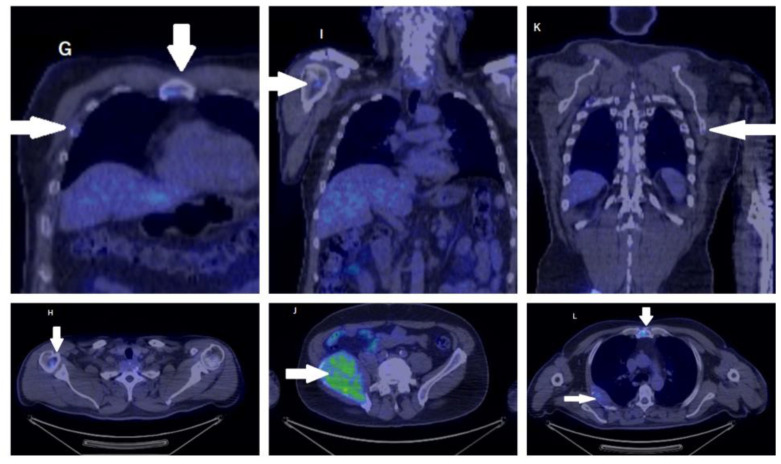
PET-CT showed an activated metabolism (arrow) of a mass in the right iliac wing of 10/7.5 cm that invaded the neighboring endo- and exopelvic structures. The mass showed an inhomogeneous uptake of FDG. Other similarly moderate FDG-capturing osteolytic lesions could be distinguished in the right posterior fourth costal arch (invasive and dimensional progression compared to the CT scan performed 1 week before), the right fourth anterior costal arch, the left sixth lateral costal arch, the sternal manubrium, the medial angle of the scapula on the left, the C7 (with a major risk of subsidence), the T7 and T9 vertebral bodies, the right lateral clavicular extremity, the right humeral head, the apex, the right temporal bone, right parietal bone, and the left sciatic tuberosity.

**Figure 5:**
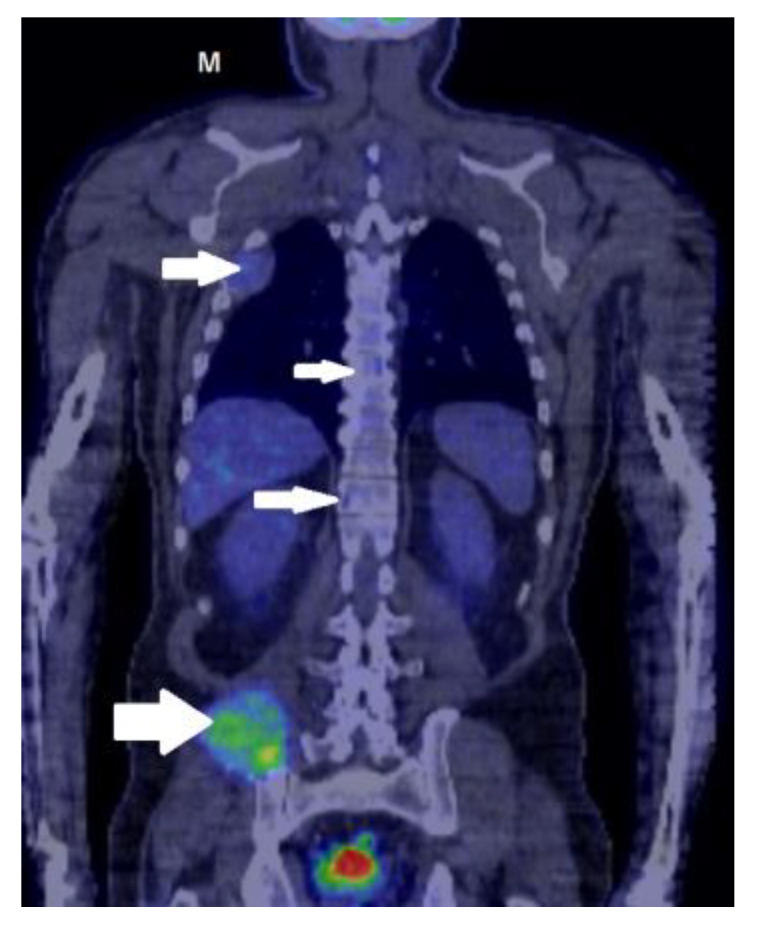
The whole-body PET-CT showed (arrow) the activated metabolism of a 10 to 7.5 cm mass in the right iliac wing that invaded the neighboring endo- and exopelvic structures. The mass showed an inhomogeneous uptake of FDG. Other similarly moderate FDG-capturing osteolytic lesions could be distinguished in T7 and T9 vertebral bodies, the right lateral clavicular extremity, and the right humeral head.

The whole-body PET-CT showed the activated metabolism of a mass in the right iliac wing measuring 10 to 7.5 cm, which invaded the neighboring endo- and exopelvic structures. The mass showed an inhomogeneous uptake of FDG. Other similarly moderate FDG-capturing osteolytic lesions could be distinguished in the right posterior fourth costal arch (invasive and dimensional progression compared to the CT scan performed 1 week before), the right fourth anterior costal arch, the left sixth lateral costal arch, the sternal manubrium, the medial angle of the left scapula, C7 (with a major risk of subsidence), T7, and T9 vertebral bodies, the right lateral clavicular extremity, the right humeral head, apex of the right temporal bone, the right parietal bone, and the left sciatic tuberosity [Figure 4 and Figure 5].

The bone marrow biopsy concluded medullary iron blockage with moderate hyperplasia of the plasmacyte, affecting 4.5% of nucleated cells.

Based on the complete blood cell counts, the protein electrophoresis, the serum electrophoresis, the immunoquantification, and the bone marrow biopsy, the diagnosis of monoclonal gammopathy of unspecified etiology of type IgG with lambda chains was established.

A bone biopsy was taken from the iliac lesion, demonstrating the diagnosis of multiple myeloma type IgG with kappa chains (CD-138 positive; kappa chains weakly intensify CD79a-positive, CD56-positive, and CD20-negative for lambda chains). The Ki37 cell proliferation index was 40%. The disease was classified as stage III according to the International Staging System (R2-ISS) for overall survival in multiple myeloma.

The patient underwent hematological treatment with daratumumab, bortezomib, thalidomide, dexamethasone, and bisphosphonate. The symptoms of bone pain were relieved. The patient is still in the follow-up stage.

## 3. Discussion

As a rare malignant tumor of the nasal cavity with a frequency of only 0.4 million per year, olfactory neuroblastoma was described for the first time in 1924 [16].

The tumor can affect both children and adults; in adults, the disease generally occurs between the fifth and sixth decades of life. Its origin is still unknown. No lifestyle risk, environmental, or geographic factors are linked to its apparition. The symptoms may vary from unilateral nasal obstruction (70%) to epistaxis (46%; see [7,9]). The gold standard of diagnosis for olfactory neuroblastoma is a biopsy and an anatomopathological examination.

Having neuroectodermal and epithelial origins, olfactory neuroblastoma presents itself as a unilateral, polypoid tumor formation of low consistency with a nonspecific clinical presentation. The olfactory epithelium can be identified in the mucosa of the superior and middle turbinates and also in the mucosa of the nasal septum [17].

The immunohistochemicalexamination is based on positive markers for S100, BCL2 [17], and PGP9.5 and negative markers for keratin, muscle, melanoma, and lymphoma.

Olfactory neuroblastoma poses a high risk of local invasion, recurrence, and distant metastasis [18]. Dulguerov et al. found in their meta-analysis that cervical lymph node metastasis is the most important prognostic factor in olfactory neuroblastoma that negatively affects survival [9].

Castelnuovo et al. reported, in a total of 10 patients treated with endoscopic surgery, the presence of cervical metastasis 21 months after surgery. The patients underwent a bilateral modified neck dissection plus radiotherapy [19]. Some studies recommend cervical neck dissection for metastases occurring 6 months or more after treatment of the primary site. Naples et al. found in a meta-analysis that elective supra-omohyoid neck dissection is a reasonable option for patients with Kadish stage B and TNM stage N0 [4,5].

Based on our experience, we think that the endonasal approach achieves a complete resection for small, localized lesions when no reconstruction is needed, and all the lesions can be resected with negative margins. Our patient was, at the time of the diagnosis, N0M0, so we did not perform a neck dissection. The endonasal excision allows rapid recovery and returnsto daily activities for the patients, improving their quality of life.

The infrequency of these tumors has limited the possibility of categorizing the prognostic factors and specific protocols of treatment. Several staging systems have been proposed. The most commonly used was proposed by Kadish et al. in 1976 and modified in 1993. Nowadays, some institutions apply the TNM staging system by the AJCC based on the Dulguerov modified version of staging [9,20,21] (Table 1).

A meta-analysis compared the outcomes of the Kadish and Dulguerov staging systems, finding that both systems correlated with prognostic factors in terms of disease-free and overall survival, with the Dulguerov system having a superior performance [21].

CT and MRI images are essential for correct staging. PET-CT can identify local recurrences and metastases. The anatomopathologicalexamination is the gold standard for diagnosis.

The typical recommended treatment for olfactory neuroblastoma consists of endoscopic surgery resection associated with radiochemotherapy. Endonasal endoscopic surgery is preferred due to its efficient local control and lower morbidity [22]. In our center, we consider and use the open approach when there is an extensive tumor with intracranial involvement or when a pericranial flap for reconstruction is needed [23].

However, in this particular case, the imaging suggests no involvement of the cribriform plate, base of the skull, orbit, or intracranial cavity. As described in the literature, endoscopic resection allows the total excision of small lesions. Due to its location in the right nasal fossa and the fact that it had not spread into the adjacent structures, we performed an endoscopic approach.

In a retrospective study by Gallia et al., eight patients with olfactory neuroblastoma treated by endonasal endoscopic surgery were identified. They had a complete resection and negative intraoperative margins, with no evidence of disease over a mean follow-up of over 27 months [3].

Newer radiotherapy techniques have been added, reducing cerebral and ocular toxicity over time [24]. Neoadjuvant chemotherapy for tumor reduction can improve surgical management by reducing the size of the tumor and its complications [25,26,27].

Due to the delayed regional recurrences associated with olfactory neuroblastoma, prolonged surveillance is recommended. We highlight in our report the aggressiveness of this tumor and the importance of including PET-CT in the monitoring follow-up protocol for olfactory neuroblastoma while also considering that distant metastases (approximately 10%) can occur irrespective of the grade of the tumor [28].

In our report, osteolytic lesions raised the suspicion of distant metastases of the olfactory neuroblastoma and multiple myeloma. Distant recurrences of olfactory neuroblastoma are described in the literature. In an article by Loy et al., 34% of patients developed recurrent disease, and the most distant metastases were osseous (humerus, lumbar spine, and diffuse bone metastases) [29]. The symptomatology of the patient correlated with theirhistory of olfactory neuroblastoma, raising the suspicion of a distant recurrence of the disease. However, the clinical ENT exam and negative biopsy of the sinus confirmed a hematological malignancy.

In multiple myeloma, malignant plasma cells proliferate in the bone marrow, displacing normal blood cells and leading to disease and symptom manifestations such as generalized weakness, weight loss, bone pain, hypercalcemia, and anemia [30].

Although our patient was diagnosed with a hematological disease and there is no correlation between multiple myeloma and olfactory neuroblastoma, it should be noted that long-term follow-up of an olfactory neuroblastoma patient is mandatory. The presence of a multidisciplinary team around the patient can identify and treat relapses, long-distance metastases, or even a hematological disease.

## 4. Conclusions

Rare sinonasal tumors present similar symptomatology, originating in a relatively small anatomical space. The patients often have a long history before their initial presentation. The treatment modalities have changed over time with the evolution of endoscopic surgery, and a multidisciplinary approach may improve the survival rate and the patient’s quality of life. Lifelong follow-up is crucial, combined with imaging surveillance, given the possibility of distant metastases occurring many years after treatment of the primary tumor or, as in our case, an early diagnosis of hematological disease.

## Figures and Tables

**Table 1:** Staging systems for olfactory neuroblastoma.

Kadish System (1976)		Kadish Modified System		TNM Staging System for Olfactory Neuroblastoma from the Dulguerov Modified Version	
Group A	Tumor confined to nasal cavity	Group A	Tumor confined to nasal cavity	T1	Tumor involving the nasal cavity and/or paranasal sinuses (excluding sphenoid), sparing the most superior ethmoidal cells
Group B	Tumor involved nasal cavity and paranasal sinuses	Group B	Tumor involved nasal cavity and paranasal sinuses	T2	Tumor involving the nasal cavity and/or paranasal sinuses (including the sphenoid) with extension to or erosion of the cribriform plate
Group C	Tumor spread beyond the nasal cavity and paranasal sinuses	Group C	Tumor extent beyond nasal cavity and paranasal sinuses, including involvement of the cribriform plate, base of the skull, orbit or intracranial cavity	T3	Tumor extending into the orbit or protruding into the anterior cranial fossa, without dural invasion
		Group D	Tumor with metastasis to cervical lymph nodes or distant sites	T4	Tumor involving the brain
				N0	No cervical lymph-node metastasis
				N1	Any form of cervical lymph-node metastasis
				M0	No metastases
				M1	Distant metastasis
